# Floor-of-mouth metastasis in colorectal cancer

**DOI:** 10.4103/0256-4947.70583

**Published:** 2011

**Authors:** Tejinder Singh, Usha Amirtham, C. T. Satheesh, K. C. Lakshmaiah, T. M. Suresh, K. Govind Babu, C. Ramachandra

**Affiliations:** a From the Department of Medical Oncology, Kidwai Memorial Institute of Oncology, Bangalore, India; b From the Department of Pathology Kidwai Memorial Institute of Oncology, Bangalore, India; c From the Department of Surgical Oncology, Kidwai Memorial Institute of Oncology, Bangalore, India

## Abstract

Colorectal cancers have potential for lymphatic and hematogenous metastases. Surgery is the definitive treatment, but the prognosis can be improved with the addition of chemotherapy, radiotherapy or both. However, the incidence of recurrence, both local and distant, remains significant. Distant metastases occur most often in the liver and lung; however, metastases to bone, adrenals, lymph nodes, brain, skin and the oral region have been reported. Metastases to the oral region are uncommon and may occur in the oral soft tissues or jaw bones. The prognosis in such patients is usually very poor. We report a case of colorectal carcinoma with metastasis to the floor of the mouth. This is probably the first reported case of metastasis to the floor of the mouth in a patient with colorectal cancer.

Metastatic spread from colorectal carcinoma is quite predictable, and is initially by the lymphatic route, followed by the hematogenous route. The commonest sites for distant metastases are liver and lung. Metastatic tumors in the oral region are uncommon and account for approximately 1% of all malignant oral tumors.^1^ Metastases in the oral region can occur in oral soft tissues or the jawbones. Metastatic tumors in the jawbones are more frequently reported than those in the oral mucosa (by a ratio of 2.5:1). The most common primary sources of metastatic tumors in the oral region are cancers in the breast, lung, kidney, bone or colorectum.^1^ We report a case of metastasis to the floor of mouth from colorectal carcinoma.

## CASE

**Figure 1:**
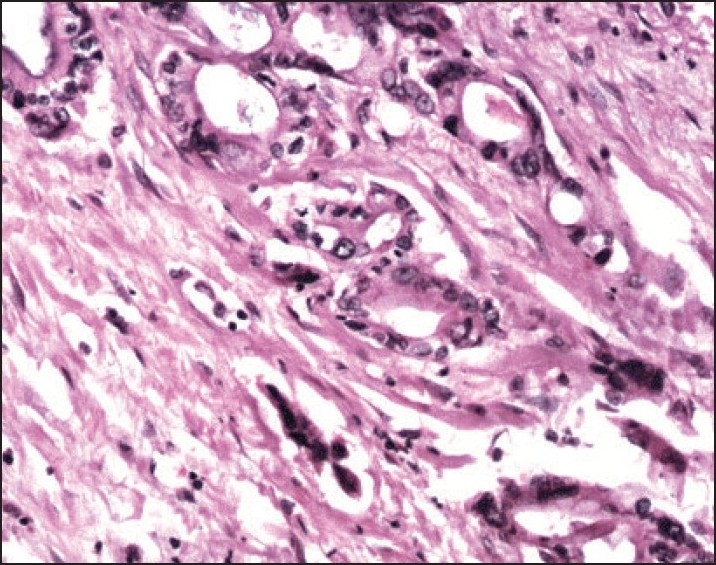
Rectal biopsy. Neoplasm composed of pleomorphic epithelial cells arranged in glandular pattern (hematolxylin and eosin ×10)

**Figure 2:**
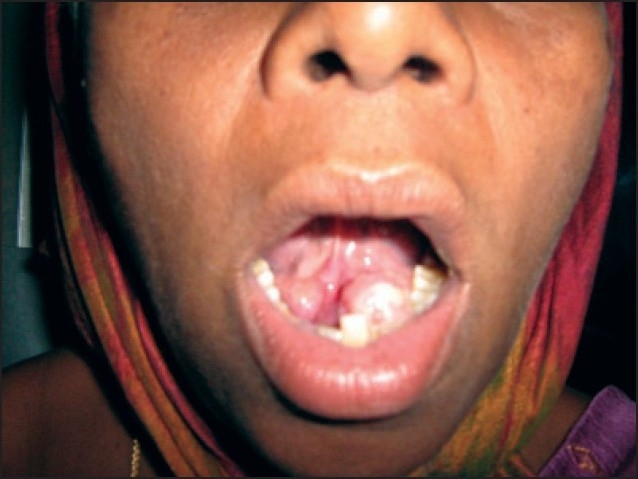
Photo of floor-of-mouth lesion.

**Figure 3:**
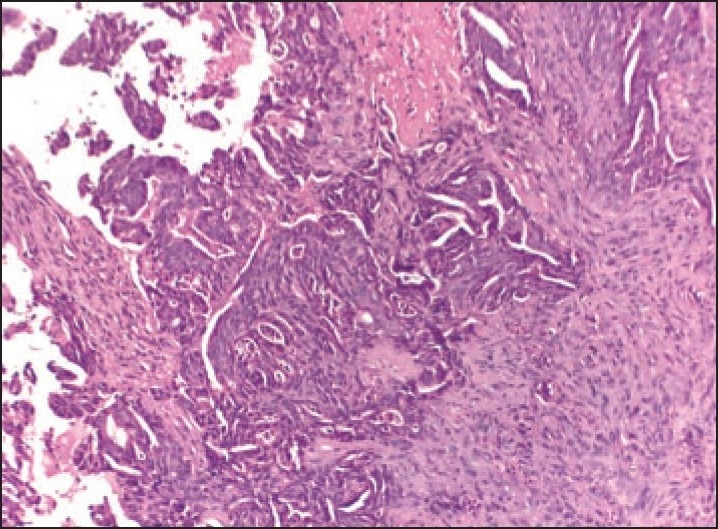
Biopsy of floor of mouth. Fibroconnective tissue with neoplastic glands (hematoxylin and eosin, ×10)

A 42-year-old woman was admitted in December 2003 with a 10-day history of blood and mucus in the stool. Rectal examination and endoscopic evaluation showed a circumferential lesion that began 4 cm above the anal verge and caused an obstruction. A CT scan of the abdomen demonstrated a mass of 4.5 cm in length with filling defect. A transanal incisional biopsy was performed. Histologic examination revealed an adenocarcinoma. The patient underwent anteroposterior resection with total mesorectal excision. Histological examination revealed a mucinous adenocarcinoma of the rectum extending through the whole muscular layer with invasion of the serosa (**Figure 1**). There was nodal involvement (two lymph nodes), and it was staged T3N1M0. Postoperatively, she received a course of radiotherapy (RT) consisting of 50 Gy in 25 fractions to the whole pelvis. Following RT, she received 6 cycles of 5-fluorouracil/leucovorin. She remained symptom free until January 2007. She was readmitted with persistent lower abdominal pain of 1-month duration. Clinical examination was noncontributory. CT scan of the abdomen revealed 3.8×3.0×3.2-cm hypodense lesion in the mid-presacral region. CT-guided fine-needle aspiration cytology of the lesion was suggestive of recurrence of adenocarcinoma. She was treated with 6 cycles of FOLFOX4 (oxaliplatin, folinic acid and 5-fluorouracil), and a CT scan of the abdomen subsequently revealed complete disappearance of the lesion. Within 2 months, she was readmitted with a growth in the floor of the mouth (**Figure 2**). A punch biopsy was performed. Histopathological examination revealed a well-differentiated adenocarcinoma (**Figure 3**), and the original primary and floor-of-mouth metastases were morphologically similar. She was treated with chemotherapy regimen consisting of FOLFOX4 with bevacizumab. Two cycles of chemotherapy elicited no response, and the growth was progressively increasing in size. Chemotherapy was stopped and she was treated with radiotherapy consisting of 64 Gy/32 Fr. She did not respond to radiotherapy either and succumbed to the disease after 20 days.

## DISCUSSION

From available evidence, it is known that patients with disseminated colorectal cancer fare poorly, which was observed in the present case. Oral metastatic tumors are uncommon and comprise approximately 1% of all malignant oral neoplasms.^1^ Most lesions are in the jawbones, and only 16% are in soft tissue, such as the gingival tissue.^2^ The breast is the most common site for cancers that metastasize to the oral soft tissues in females (**Table 1**). Few cases of metastasis to the oral cavity from colorectal carcinoma have been reported in literature (**Table 2**). In most cases, the site of metastasis is gingival. Our case is unique in that probably this is the first case report of metastasis to the floor of mouth from colorectal carcinoma.

**Table 1:** Common primary tumor sites for cancers that metastasize to the oral soft tissues.

Breast	24%
Genital organs	17%
Lung	12%
Kidney	10%
Bone	10%
Skin	7%
Rare tumors	20%

**Table 2:** Reports of metastasis to oral cavity from colorectal carcinoma.

	Author	Site of metastasis
1	David Moffat et al^3^	Gingiva
2	Rusthuven et al^4^	Tooth socket
3	Alvarez et al^5^	Gingiva
4	Morimasa et al^6^	Gingiva
5	Masahiko et al^7^	Gingiva
6	Yoshihiro et al^8^	Gingiva
7	Rentschler et al^9^	Gingiva

Whereas early detection and surgery remain the main therapeutic options for adenocarcinoma of the colon, a good response to chemotherapy in an advanced case is also reported.^10^ Metastatic lesions in the oral cavity cause acute progressive discomfort, such as pain or bleeding, as in this case. Therefore, even in cases with advanced malignant disease, palliative treatment is necessary to improve the quality of life. Early diagnosis and treatment are essential to prevent the pain and discomfort associated with ulceration, infection and local tissue destruction by such lesions.^10^ Although local lymph nodes, liver and lungs are the common and initial sites of spread from colorectal cancers, disseminated metastases with sparing of these organs is unlikely but possible. Combination chemotherapy, as mentioned, might be the ideal regimen, although the prognosis remains dismal. The possibility that a floor-of-mouth growth represents a metastatic neoplasm must be considered in patients with a known or suspected malignancy such as colorectal carcinoma.
